# Exosomal miR‐199a‐3p Secreted From Cancer‐Associated Adipocytes Promotes Pancreatic Cancer Progression

**DOI:** 10.1002/cam4.70265

**Published:** 2024-10-21

**Authors:** Kazuyoshi Noda, Yasushi Sato, Yasuyuki Okada, Kensei Nishida, Yutaka Kawano, Toshihito Tanahashi, Masahiro Bando, Koichi Okamoto, Masanori Takehara, Masahiro Sogabe, Hiroshi Miyamoto, Kei Daizumoto, Hiroomi Kanayama, Tetsuji Takayama

**Affiliations:** ^1^ Department of Gastroenterology and Oncology Tokushima University Graduate School of Biomedical Sciences Tokushima Japan; ^2^ Department of Community Medicine for Gastroenterology and Oncology Tokushima University Graduate School of Biomedical Sciences Tokushima Japan; ^3^ Department of Pathophysiology Tokushima University Graduate School of Biomedical Sciences Tokushima Japan; ^4^ Department of Urology Tokushima University Graduate School of Biomedical Sciences Tokushima Japan

**Keywords:** biomarker, cancer‐associated adipocytes, miR‐199a‐3p, pancreatic ductal adenocarcinoma

## Abstract

**Background:**

Pancreatic ductal adenocarcinoma (PDAC) is a highly aggressive cancer. Recent studies indicated that cancer‐associated adipocytes (CAAs) play crucial roles in tumor progression; however, the precise mechanism remains unknown. Here, we analyzed specific exosomal microRNAs (miRNA) signatures derived from pancreatic CAAs to investigate their role in cancer progression.

**Methods:**

CAAs were generated by co‐culturing human adipocytes with human pancreatic cancer cells, and exosomes were isolated from the CAA‐conditioned medium (CAA‐CM). Small RNA‐seq analysis was used to identify differentially expressed miRNAs in these exosomes. The effects of miRNAs on cell proliferation, migration/invasion, and drug sensitivity were examined. Luciferase reporter assays, real‐time polymerase chain reaction, and western blotting were performed to investigate the molecular mechanisms of the miRNAs. The clinical relevance of the miRNAs was investigated using publicly available data and our cohort of patients with PDAC.

**Results:**

miR‐199a‐3p expression was significantly increased in CAA‐CM‐derived exosomes. CAA‐derived exosomes transferred miR‐199a‐3p to pancreatic cancer cells. Transfection with miR‐199a‐3p increased the proliferation, invasion, migration, and drug resistance of pancreatic cancer cells by downregulating SOCS7, increasing STAT3 phosphorylation, and upregulating SAA1 expression. High tissue miR‐199a‐3p expression is correlated with poor prognosis in patients with PDAC. Liquid biopsies revealed that exosomal miR‐199a‐3p could accurately differentiate patients with PDAC from healthy controls. Multivariate survival analysis indicated that miR‐199a is an independent prognostic factor for PDAC.

**Conclusion:**

miR‐199a‐3p in CAA‐derived exosomes contributes to the malignant transformation of pancreatic cancer via the SOCS7/STAT3/SAA1 pathway, suggesting its potential as a biomarker and therapeutic target for PDAC.

## Graphical abstract

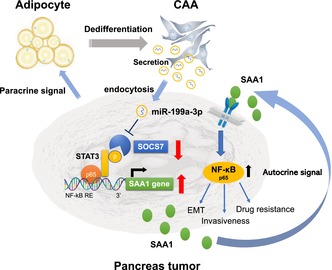

## 1 Introduction

Pancreatic cancer is highly lethal and ranks third in global cancer‐related deaths [1]. Pancreatic ductal adenocarcinoma (PDAC), a subtype of pancreatic cancer, is known for its aggressive nature, rapid metastasis, and resistance to treatment. It carries a grim prognosis, with a survival rate of less than 10% beyond 5 years [2]. Therefore, it is necessary to urgently establish effective therapeutic strategies and reliable biomarkers for the early diagnosis of PDAC.

Epidemiological studies have demonstrated that obesity increases the risk of pancreatic cancer [3, 4]. Clinicopathological analyses have revealed that pancreatic intralobular fat infiltration is a risk factor for precancerous pancreatic lesions [5]. Furthermore, peripancreatic fat invasion predicts poor prognosis after PDAC surgery [6]. EL‐KrasG12D/PEDF‐deficient mice showed increased peripancreatic fat levels associated with PDAC cell invasion [7]. Similarly, mice with conditional KrasG12D mutations fed a high‐fat diet showed more precancerous lesions and invasive carcinomas [8], strongly suggesting the tumor‐promoting role of adipose tissue in the PDAC microenvironment. Abundant stromal cells, including cancer‐associated fibroblasts (CAFs), tumor‐associated macrophages, pancreatic stellate cells, immune cells, endothelial cells, adipocytes, and extracellular matrix proteins, are also present in this microenvironment [9], significantly impacting PDAC progression [10]. However, the precise interactions between adipocytes and cancer cells in the PDAC microenvironment remain unknown.

Accumulating evidence confirms that tumor cells can significantly affect the surrounding adipocytes in various cancers [11]. Specifically, adipocytes neighboring invasive cancer cells exhibit altered characteristics, including a fibroblast‐like phenotype with a smaller size, dispersed lipid droplets, and reduced expression of differentiation markers. Although factors that dictate or promote the dedifferentiation of adipocytes into a fibroblastic phenotype remain unidentified, these adipocytes are termed cancer‐associated adipocytes (CAAs) [12, 13, 14, 15]. We have previously shown that murine 3T3‐L1 adipocytes co‐cultured with pancreatic cancer cells undergo dedifferentiation into CAAs, adopting a fibroblast‐like appearance with decreased lipid content and downregulated adipocyte markers [16].

CAAs are key players in malignant tumor progression [17, 18, 19]. CAAs modify the behavior of breast, colon, prostate, and ovarian tumors, resulting in heightened aggressiveness characterized by increased proliferation, invasion, chemotherapy resistance via adipokine secretion, and modulation of cancer cell metabolism [17, 18, 19]. Nonetheless, the precise molecular mechanisms underlying this phenomenon remain unclear. Our previous research demonstrated that the conditioned medium from CAAs (CAA‐CM) enhances migration, invasion, and chemoresistance and promotes epithelial‐mesenchymal transition in pancreatic cancer cells by elevating SAA1 expression. Additionally, we found that SAA1 expression was significantly correlated with the survival rate and was an independent prognostic marker for patients with PDAC. These findings suggest that CAA‐derived factors drive pancreatic cancer progression via SAA1 upregulation [16]. However, the specific factors within CAA‐CMs responsible for regulating SAA1 expression remain unidentified.

Exosomes are small membranous vesicles (30–150 nm) containing a lipid bilayer that play a crucial role in regulating gene expression and biological function [20]. They transfer bioactive molecules, such as mRNA, microRNAs (miRNAs), proteins, and lipids, to their recipient cells. miRNAs are 18–22 nucleotide‐long RNA molecules that modulate gene expression by binding to their complementary 3′ untranslated regions (UTRs) and promoting mRNA degradation [21]. MiRNAs regulate cell proliferation, differentiation, metabolism, and apoptosis [22]. Exosome‐mediated miRNA transfer is particularly likely because exosomal miRNAs are more stable in circulation. Exosomal miRNAs have been reported to contribute to responsiveness to therapy, prognosis, and tumorigenesis in multiple tumor types [23, 24].

Adipose tissue serves as a significant source of circulating exosomal miRNAs [25]. However, the specific exosomal miRNA signature of CAAs and their functional role in modulating the phenotype of recipient cancer cells have not been explored. Hence, this study employed RNA‐seq analysis to examine the miRNA profile of exosomes released in CAA‐conditioned media to unravel the regulatory mechanisms underlying SAA1 expression. Moreover, we investigated the clinical significance of miR‐199a‐3p expression in liquid biopsies from patients with PDAC.

## 2 Material and Methods

### 2.1 Pancreatic Cancer Cell Lines and Culture Conditions

Panc‐1 human pancreatic adenocarcinoma cells were obtained from the American Type Culture Collection (ATCC). PK1 and PK‐45H cells were obtained from the RIKEN Cell Bank. Panc‐1 cells were cultured in Dulbecco's Modified Eagle's Medium (DMEM) supplemented with 10% fetal bovine serum (FBS) at 37°C in a 5% CO_2_ environment. PK‐1 and PK‐45H cells were cultured in Roswell Park Memorial Institute 1640 medium (RPMI‐1640) supplemented with 10% FBS under the same conditions (37°C in 5% CO_2_).

### 2.2 Isolation of Adipocytes From Fat Tissues of Patients With Benign Diseases

Adipose tissue was obtained from the retroperitoneal fat of male patients diagnosed with benign urological diseases. The tissue was minced into small pieces, rinsed once with saline, and centrifuged at 380 × *g* for 5 min. Subsequently, the tissues were transferred into isolation medium comprising DMEM supplemented with 20% FBS, 0.5% penicillin, 0.5% streptomycin, and 0.06% collagenase, followed by digestion at 40°C for 40 min with continuous agitation. The suspended cells were then filtered through a 70 μm nylon filter and centrifuged at 300 × *g* for 5 min.

The resulting cells, at a concentration of 2 × 10^5^ cells/mL, were resuspended in 10 mL of isolation medium without collagenase. They were seeded in a 10‐cm dish and incubated overnight at 37°C in a 5% CO_2_ environment. On the subsequent day, the adherent cells were washed with phosphate buffered saline (PBS), and the culture medium was changed to a fresh isolation medium without collagenase. The cells were cultured at 37°C in 5% CO_2_ for 7 days to induce preadipocytes. These preadipocytes were further differentiated into mature adipocytes by incubating them in a differentiation medium (DM‐2; Zen‐Bio, NC, USA) for 3 days, followed by a maintenance medium (AM‐1; Zen‐Bio, NC, USA) for 14 days, adhering to the manufacturer's protocol.

Human mature adipocytes were subjected to Oil Red O staining, and the retained dye within the cells was eluted using isopropanol. The lipid content was quantified by measuring the absorbance at 496 nm. Ethical approval was obtained from Tokushima University Hospital's ethics committee, and all patients provided written informed consent for the use of their clinical specimens in this study (No. 2222).

### 2.3 Induction of Cancer‐Associated Adipocytes

To induce CAAs, we employed a Transwell culture system (ThinCert 657641, Greiner Bio‐One) as previously outlined [16]. In brief, Panc‐1 cells were seeded in the upper chamber of a Transwell (1 × 10^5^ cells/well), while 1 × 10^4^ mature adipocytes were seeded in the lower chamber. The cells were cultured in DMEM supplemented with 10% FBS at 37°C in a 5% CO_2_ environment. Half of the co‐culture medium was replaced every 3 days for a duration of 2 weeks.

### 2.4 Preparation of the Conditioned Medium

To prepare the conditioned medium (CM), CAA (CAA‐CM) or adipocyte‐conditioned medium (A‐CM) were cultured in exosome‐depleted media containing 10% FBS (EXO‐FBS‐50A‐1, SBI, CA, USA) for 24 h. Subsequently, the used medium was collected and filtered with 0.22‐μm filters (Millex, Millipore, MA, USA) to remove the dead or floating cells, stored at 4°C, and used within 1 month.

### 2.5 Exosome Isolation

Exosomes (small extracellular vesicles per the Minimal Information for Studies of Extracellular Vesicles [MISEV] 2018 nomenclature) were isolated from the CM following MISEV 2018 guidelines established by the International Society for Extracellular Vesicles [20]. Briefly, both A‐CM and CAA‐CM were centrifuged twice at 3000 × *g* for 10 min at 4°C to remove debris. The resulting supernatant was then centrifuged at 100,000 × *g* for 90 min to pellet the exosomes. These exosomes were subsequently washed with sterile PBS and purified through ultracentrifugation at 100,000 × *g* for 90 min. Finally, the exosomes were resuspended in 100–500 μL of PBS, which had been filtered through 0.22 μm filters (Millex, Millipore, MA, USA). Subsequently, the exosomes underwent NanoSight particle tracking analysis (NanoSight NS300; Malvern Instruments, Malvern, UK) to determine nanoparticle concentrations and size distribution. Furthermore, a bicinchoninic acid protein assay (Thermo Fisher Scientific, Waltham, MA, USA) was used to measure protein concentration. The isolated exosomes were then stored at 4°C until further use.

### 2.6 MiRNA Transfection

MiRNA mimics and random miRNAs (as controls) were purchased from Thermo Fisher Scientific. Each miRNA (10 nM) was transfected into cells using the Lipofectamine RNAiMAX Reagent (Life Technologies), as previously described [26].

### 2.7 Quantitative RT‐PCR Analysis

Total RNA was extracted from cells or exosomes using an RNeasy Mini Kit (Qiagen, Germany), and complementary DNA was synthesized using a TaqMan microRNA reverse transcription kit (Thermo Fisher Scientific) following the manufacturer's instructions. The quantity and quality of the total RNA were assessed using a NanoDrop spectrophotometer (Thermo Fisher Scientific). Reverse transcription‐quantitative PCR (RT‐qPCR) was conducted as previously described [16]. Pre‐designed TaqMan Gene Expression Assays (Applied Biosystems) were performed with High‐Capacity RNA‐to‐cDNA Kit (Applied Biosystems) and TaqMan miRNA reverse transcription kits (Thermo Fisher Scientific). *GAPDH* (Hs02758991_g1), *SAA1* (Hs07291672_g1), *SOSC7* (Hs00322554_m1), U6 (001973, as an internal control), hsa‐miR‐199a‐3p (002304), hsa‐miR‐6126 (475618_mat) were employed in this study.

### 2.8 MiRNA Target Prediction

MiRNA target prediction and analysis were performed using the TargetScan algorithm (http://www. targetscan.org).

### 2.9 MiRNA Expression in Pancreatic Tissues

The expression profiles of miRNAs in pancreatic tissues were acquired from the Genomic Data Commons data portal [27].

### 2.10 Patient Cohorts

A total of 61 patients with PDAC and 10 healthy donors (HDs) were recruited at Tokushima University Hospital in Tokushima, Japan, between October 2020 and April 2022. Patients with PDAC were stratified according to the Union for International Cancer Control (UICC) TNM classification (8th Edition). None of the HDs had a history of malignancy. Patients who had been administered neoadjuvant chemotherapy or radiotherapy were excluded to eliminate potential variations in miRNA expression. The clinicopathological characteristics of the included patients and donors are summarized in Table S1.

### 2.11 Statistical Analysis

The *t*‐test was performed using GraphPad Prism software (version 8.0; GraphPad, CA, USA) to establish statistical significance. Kaplan–Meier plots were generated for survival analyses, and statistical differences were assessed using the log‐rank (Mantel–Cox) test. Progression‐free survival (PFS) was defined as the time from the start of treatment to the first occurrence of disease recurrence or death from any cause. Overall survival (OS) was defined as the time interval between the treatment initiation date and cancer‐related death. Patients who were still alive without recurrent disease or were lost to follow‐up at the time of analysis were censored at their last follow‐up date. Fisher's exact test was applied to compare clinicopathological parameters. The diagnostic potential of exosomal miR‐199a‐3p was evaluated by calculating the area under the receiver operating characteristic curve (AUC) and its corresponding 95% confidence interval (CI). The Cox proportional hazards regression model was employed to assess the prognostic value of miR‐199a‐3p in serum exosomes. Statistical significance was defined as *p* ≤ 0.05. More details about the Methods are provided in the Supporting Information (Data S1).

## 3 Results

### 3.1 Extensive Phenotypical Changes in Human Adipocytes Following Co‐Culture With Pancreatic Cancer Cells

To investigate the effects of pancreatic cancer cells on human adipocytes in vitro, we employed an indirect co‐culture system. Human adipocytes co‐cultured with Panc‐1 cells exhibited an elongated fibroblast‐like morphology, which was distinct from that of adipocytes cultured with DMEM and 10% FBS alone (Figure S1A). Furthermore, we observed a significant reduction in the number of lipid droplets in co‐cultured adipocytes compared to that in adipocytes cultured alone, as confirmed by lipid content quantification (Figure S1B). These findings support the induction of CAAs from human adipocytes, as previously reported [16].

### 3.2 Effects of CAA‐CM on Panc‐1 Cell Proliferation, Migration, Invasion, and Chemotherapy Resistance

Next, we investigated the influence of CAA‐derived factors on the behavior of Panc‐1 cells, focusing on their proliferation, migration, invasion, and resistance to gemcitabine. The results revealed a substantial increase in Panc‐1 cell proliferation when exposed to CAA‐CM compared to when exposed to adipocyte‐conditioned medium (A‐CM) and control media (Figure S1C). We also conducted a scratch wound assay to assess cell migration, with representative images depicting wound healing in Panc‐1 cells co‐cultured with CAA‐CM, A‐CM, and control medium (Figure S1D). Quantitative analysis demonstrated a significantly larger migrated area in Panc‐1 cells treated with CAA‐CM than that in Panc‐1 cells treated with A‐CM and the control medium (Figure S1E). Furthermore, we performed an invasion assay, and representative images are displayed in Figure S1F. Quantitative analysis revealed a markedly higher cell invasion rate following co‐culture with CAA‐CM compared to those of co‐cultures with A‐CM and the control medium (Figure S1G). To evaluate gemcitabine sensitivity, Panc‐1 cells were assessed, and the results indicated that cells cultured in CAA‐CM exhibited greater resistance to gemcitabine than those cultured in A‐CM and the control medium (Figure S1H). Consistent with our previous findings regarding the promotion of malignancy by CAA‐CM [16], SAA1 expression in Panc‐1 cells was significantly upregulated in the presence of CAA‐CM compared to that in A‐CM and the control media (Figure S1I).

### 3.3 CAA‐CM‐Derived Exosomes Revealed Typical Exosome Characteristics

**FIGURE 1:**
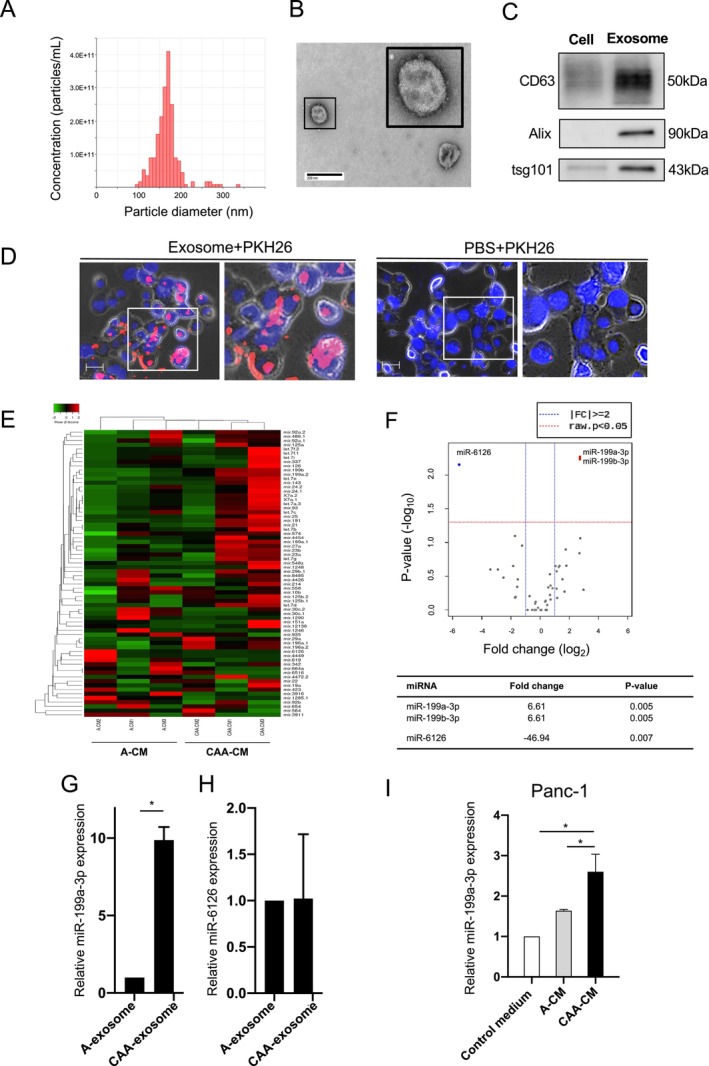
Identification of differentially expressed miRNAs in CAA‐CM‐derived exosomes using next‐generation sequencing analysis. (A–C) Characterization of serum exosomes. (A) Nanoparticle tracking analysis of the size distribution and number of exosomes derived from the cancer‐associated adipocytes‐conditioned medium (CAA‐CM) isolated via ultracentrifugation. (B) Representative transmission electron microscopy images of CAA‐CM‐derived exosomes. Scale bar, 100 nm. (C) Western blotting analysis of exosome markers CD63, Alix, and tsg101 in CAA‐CM‐derived exosomes and exosome‐depleted supernatants of Panc‐1 cells. (D) Representative fluorescent microscopy images of the internalization of fluorescently labeled exosomes in Panc‐1 cells. Scale bar, 50 μm. (E) Hierarchical clustering analysis of differentially expressed miRNAs in CAA‐CM‐ and adipocytes‐CM (A‐CM)‐derived exosomes. The heatmap shows the top 54 differentially expressed miRNAs between the two groups. Each row represents a single miRNA and each column represents an individual sample. (F) Volcano plot of the miRNAs of 6 samples (3 CAA‐CMs and 3 A‐CMs). The abscissa represents the log_2_ (fold change) of the miRNA expression, and the ordinate represents the logarithmic transformation of the p‐value gained using the *t*‐test. (G) RT‐qPCR analysis of miR‐199a‐3p expression in adipocyte‐derived exosomes (A‐exosomes) or CAA‐derived exosomes (CAA‐exosomes). *p < 0.01. (H) RT‐qPCR analysis of miR‐6126 expression in A‐exosomes or CAA‐exosomes. (I) RT‐PCR analysis of miR‐199a‐3p expression in Panc‐1 cells cultured in the control medium, A‐CM, or CAA‐CM for 48 h. *p < 0.01.

To investigate the potential impact of CAA‐secreted exosomal miRNAs on pancreatic cancer progression, we isolated exosomes from CAA‐CM (CAA‐exosomes) and A‐CM (A‐exosomes) using ultracentrifugation. NanoSight analysis confirmed the size of the exosomes to be 100–200 nm (Figure 1A, Table S2). Transmission electron microscopy revealed their characteristic double‐layered membranes and cup‐shaped structures (Figure 1B). Western blot analysis validated the expression of exosomal markers, including CD63, Alix, and tsg101 (Figure 1C). These findings confirmed the successful isolation of exosomes from the CM.

To confirm the uptake of CAA‐exosomes by Panc‐1 cells, we labeled CAA‐exosomes with the fluorescent dye PKH26 and introduced them into the culture medium of Panc‐1 cells. Fluorescence microscopy revealed red fluorescence signals in Panc‐1 cells treated with CAA‐exosomes. In contrast, no fluorescence signals were observed in the PBS‐treated cells (Figure 1D), indicating that PKH26‐labeled exosomes were successfully internalized by the Panc‐1 cells.

### 3.4 MiR‐199a‐3p Is Upregulated in CAA‐CM‐Derived Exosomes

Next‐generation sequencing of miRNAs was performed to investigate the effect of exosomes on pancreatic cancer progression. Differentially expressed miRNAs in CAA‐exosomes and A‐exosomes were identified, revealing 54 miRNAs of interest. Hierarchical clustering revealed distinct miRNA signatures between CAA‐CMs and A‐CMs, as shown in the heatmap in Figure 1E. Volcano plots demonstrated the upregulation of miR‐199a‐3p and miR‐199b‐3p, along with the downregulation of miR‐6126 in CAA‐exosomes compared to A‐exosomes (Figure 1F). MiR‐199a‐3p, with the same mature sequence as miR‐199b‐3p [28], was chosen for further experiments. RT‐qPCR analysis validated the higher expression of miR‐199a‐3p in CAA‐exosomes than in A‐exosomes, confirming the RNA‐seq data (Figure 1G). In contrast, the expression level of miR‐6126 did not differ between CAA‐exosomes and A‐exosomes (Figure 1H). Subsequently, we explored the impact of CAA‐CM on miR‐199a‐3p levels in Panc‐1 cells. MiR‐199a‐3p expression was significantly higher in Panc‐1 cells treated with CAA‐CM than in those treated with A‐CM and the control media (Figure 1I). These findings suggest that CAA‐derived exosomes were internalized by Panc‐1 cells, leading to increased expression of miR‐199a‐3p.

### 3.5 MiR‐199a‐3p Overexpression Enhances Proliferation, Migration, Invasion, and Chemotherapy Resistance in Pancreatic Cancer Cells

**FIGURE 2:**
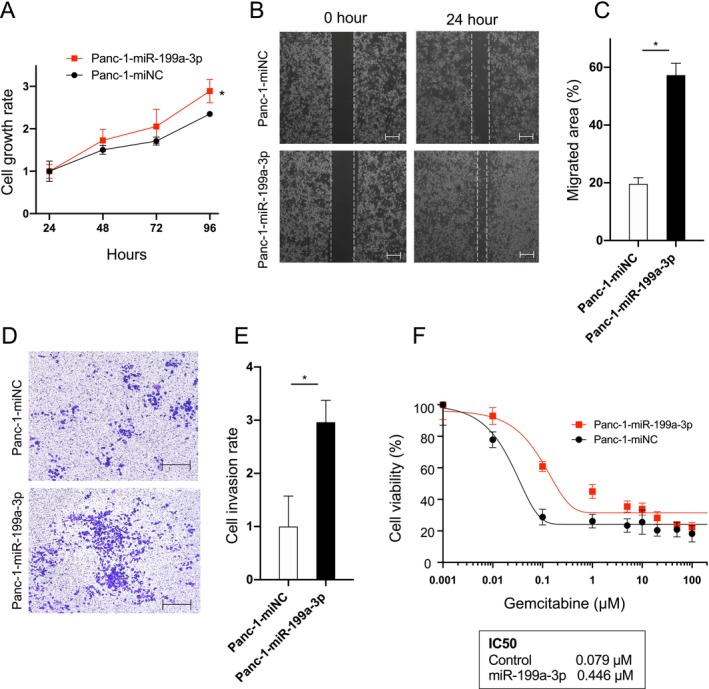
MiR‐199a‐3p promotes the growth, migration/invasion, and drug resistance of pancreatic cancer cells. (A) Growth curve of Panc‐1 cells transfected with miR‐199a‐3p mimics (Panc‐1‐miR‐199a‐3p) and scramble siRNA (Panc‐1‐miNC), as analyzed using a WST‐8 assay (*n* = 6). (B) Migration area of Panc‐1‐miR‐199a‐3p‐ or Panc‐1‐miNC‐transfected cells, as assessed using a wound healing assay. (C) Migration area was quantified using Tscratch software (*n* = 6). Scale bars, 100 μm. (D) Invasion capability of Panc‐1‐miR‐199a‐3p‐ or Panc‐1‐miNC‐transfected cells, as assessed using a Transwell invasion assay. (E) Cell migration was quantified by counting the number of migrating cells in six randomly chosen visual fields (*n* = 6). Scale bar, 200 μm. (F) Panc‐1‐miR‐199a‐3p‐ or Panc‐1‐miNC‐transfected cells were treated with different concentrations of gemcitabine for 72 h. Viable cells were quantified using a WST assay (*n* = 6). *p < 0.05.

To investigate the function of miR‐199a‐3p on cell growth and metastasis, we transfected Panc‐1 cells with either a miR‐199a‐3p mimic (Panc‐1‐miR‐199a‐3p) or a negative control sequence (Panc‐1‐miNC). Successful overexpression of miR‐199a‐3p in Panc‐1 cells was confirmed (Figure S2). Cell proliferation assays revealed a significant enhancement in cell growth in Panc‐1‐miR‐199a‐3p compared to that in Panc‐1‐miNC (Figure 2A). Additionally, we performed a scratch wound assay to evaluate cell migration. Representative images in Figure 2B clearly illustrate increased cell migration in Panc‐1‐miR‐199a‐3p compared to that in Panc‐1‐miNC (Figure 2C). An invasion assay was also conducted, and representative images are presented in Figure 2D. Quantitative analysis demonstrated a notable increase in the number of invading cells in Panc‐1‐miR‐199a‐3p compared to that in Panc‐1‐miNC (Figure 2E). Moreover, miR‐199a‐3p overexpression significantly elevated gemcitabine resistance in Panc‐1 cells, as indicated by the higher IC50 value in Panc‐1‐miR‐199a‐3p‐treated cells (Figure 2F). These findings underscore the role of miR‐199a‐3p in promoting migration, invasion, and gemcitabine resistance in Panc‐1 cells.

### 3.6 SOCS7 Was Identified as a Downstream Target of miR‐199a‐3p

**FIGURE 3:**
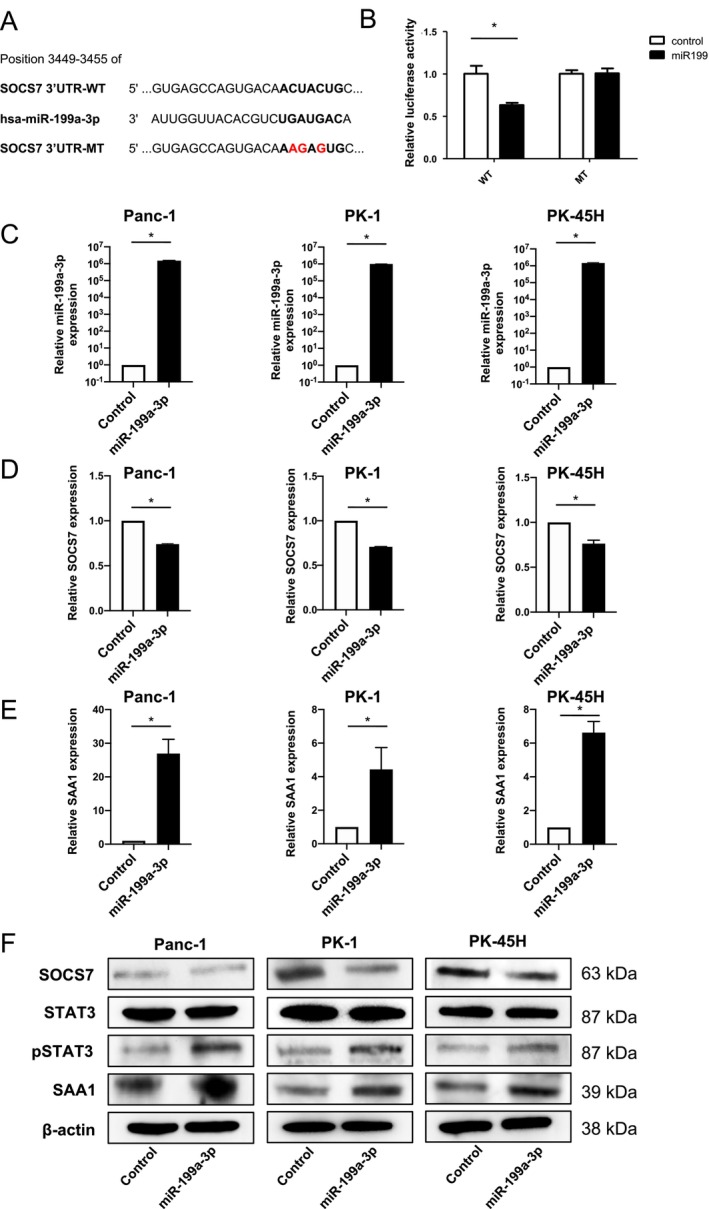
MiR‐199a‐3p suppresses SOCS7 to increase STAT3 activation and induce SAA1 expression in pancreatic cancer cells. (A, B) *SOCS7* is a downstream target gene of miR‐199a‐3p. (A) The wild‐type (WT) SOCS7 3′‐untranslated region (UTR) has a putative binding site for miR‐199a‐3p (position 3449–3455) predicted using the Target Scan database. SOCS7 3′‐UTR‐mutant type (MT) contains mutated sequences in the putative binding site of miR‐199a‐3p (shown in red). The indicated sequences (SOCS7 3′‐UTR‐WT and SOCS7 3′‐UTR‐MT) were cloned in the psiCHECK2 vector. (B) Panc‐1 cells were co‐transfected with the control miRNA mimics and psiCHECK2 vectors containing the WT or MT sequences. Luciferase activities in these cells were measured using a Dual‐Luciferase Reporter Assay System (*n* = 3). *p < 0.05. (C–E) Relative mRNA levels of various genes in Panc‐1, PK‐1, and PK‐45H cells transfected with miR‐199a‐3p mimics or control miRNA were determined via RT‐qPCR: (C) *miR‐199a‐3p*, (D) *SOCS7*, and (E) *SAA1*. *n* = 6. *p < 0.05. (F) The expression of SOCS7, STAT3, p‐STAT3 (Tyr705), and SAA1 in Panc‐1, PK‐1, and PK‐45H cells were examined using western blot analysis 24 h after transfection with miR‐199a‐3p mimics or control miRNAs.

To elucidate the regulatory mechanism by which miR‐199a‐3p upregulates SAA1 expression in pancreatic cancer, we conducted TargetScan analysis to identify potential downstream targets of miR‐199a‐3p. Among the genes identified, SOCS7, which negatively regulates STAT3 expression and interacts with the SAA1 promoter, was chosen as a candidate. TargetScan analysis predicted a matching sequence in the 3′‐UTR of *SOCS7* for miR‐199a‐3p (Figure 3A). Dual‐luciferase assays using chimeric RNAs containing the Renilla luciferase sequence and the SOCS7 3′‐UTR confirmed that miR‐199a‐3p overexpression significantly reduced Renilla_SOCS7‐3′‐UTR luciferase activity (Figure 3B). A mutant vector featuring point mutations in the miR‐199a‐3p binding sites had no effect on luciferase activity (Figure 3B). To further validate the influence of miR‐199a‐3p on SOCS7 and SAA1 expression in pancreatic cancer cells, we transfected three pancreatic cancer cell lines (Panc‐1, PK‐1, and PK‐45H cells) with a miR‐199a‐3p mimic. Overexpression of miR‐199a‐3p in each cell line was confirmed (Figure 3C), and RT‐qPCR analysis revealed a significant decrease in SOCS7 expression (Figure 3D), accompanied by a significant increase in SAA1 expression (Figure 3E). Western blot analysis of each miR‐199a‐3p‐overexpressing cell line demonstrated decreased SOCS7 expression, increased STAT3 phosphorylation, and heightened SAA1 expression (Figure 3F). These results collectively signify that miR‐199a‐3p enhances SAA1 expression in pancreatic cancer by directly targeting SOCS7 and activating STAT3.

To substantiate the importance of the SOCS7/STAT3/SAA1 pathway for miR‐199a‐3p action, we introduced a siRNA for STAT3 (*siSTAT3*) into miR‐199‐transfected Panc‐1 cells and assessed its effect on malignant transformation. A significant decrease in cell proliferation, invasiveness, and drug resistance was observed in the *siSTAT3* group compared with those in the control siRNA group (Figure S3). These results strongly suggest that miR‐199a‐3p predominantly regulates SAA1 expression through the SOCS7/STAT3/SAA1 pathway.

### 3.7 MiR‐199a‐3p Was Highly Expressed in Pancreatic Cancer Tissues

To evaluate the expression patterns of miR‐199a‐3p in different components of PDAC tissues, we performed in situ hybridization (ISH) on serial sections of pancreatic cancer tissue samples. Treatment with a miR‐199a‐3p probe showed strong miR‐199a‐3p staining in the CAAs, tumor cells, and the tumor‐stromal interface around the invasion front compared to serial sections treated with the control scrambled probe (Figure S4).

Furthermore, immunohistochemical examination of SOCS7 and pSTAT3 on consecutive sections of miR‐199‐positive PDAC tissue showed weak SOCS7 expression and relatively strong nuclear pSTAT3 expression in tumor cells (Figure S5). This result supports the notion that miR‐199a‐3p secreted from CAAs was transferred into PDAC cells by exosomes and is upregulated in PDAC through the SOCS7/STAT3 pathway.

### 3.8 Tissue miR‐199a‐3p Expression Was Negatively Correlated With Survival in Patients With PDAC

**FIGURE 4:**
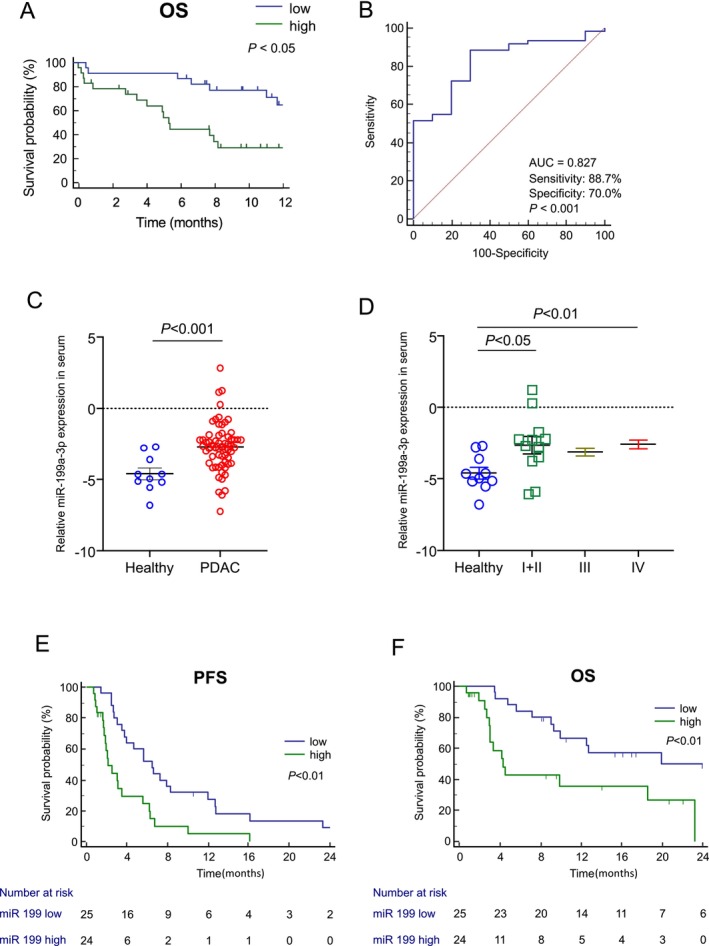
MiR‐199a‐3p expression is negatively correlated with survival in patients with PDAC. (A) Kaplan–Meier survival curves showing the overall survival of the low‐miR‐199a‐3p and high‐miR‐199a‐3p groups from the GDC TCGA Pancreatic Cancer (PAAD) dataset. The fragments per kilobase million with upper quantile (FPKM–UQ) values normalized using RNA‐seq counts were used to perform survival analyses. The cutoff value was set at 50,000 FPKM–UQ based on the median miRNA expression. Cases with ≥ 50,000 and < 50,000 FPKM–UQ were designated the high and low expression groups, respectively. (B) Receiver operating characteristic (ROC) curve analysis for discriminating between healthy individuals and patients with pancreatic ductal adenocarcinoma (PDAC). (C) Comparison of miR‐199a‐3p expression levels between healthy individuals (*n* = 10) and patients with PDAC (*n* = 61). (D) Comparison of miR‐199a‐3p expression levels in healthy individuals (*n* = 10) and patients with stages I + II (*n* = 12), III (*n* = 13), and IV (*n* = 36) PDAC. (E, F) Kaplan–Meier survival curves for (E) OS and (F) PFS in the low‐miR‐199a‐3p and high‐miR‐199a‐3p groups of patients with stage III/IV PDAC who received chemotherapy (*n* = 49). OS, overall survival; PFS, progression‐free survival.

To explore the clinical significance of miR‐199a‐3p expression in patients with PDAC, we examined its relationship with OS in stage III and IV PDAC using the pancreatic cancer dataset from the Genomic Data Commons and The Cancer Genome Atlas (TCGA). Out of the 224 patients in the dataset, miR‐199a‐3p data were accessible for 65 patients. These individuals were categorized into two groups: the low‐miR‐199a‐3p group (*n* = 32) and the high‐miR‐199a‐3p group (*n* = 33) (Figure 4A). Kaplan–Meier analysis revealed that the high‐miR‐199a‐3p group exhibited significantly shorter survival compared to the low‐miR‐199a‐3p group (12.8 months vs. not reached; hazard ratio (HR), 2.24; 95% CI: 1.03–4.88; *p* < 0.05). Similarly, based on tissue SOCS7 expression data obtained from TCGA dataset, the high‐SOCS7 group exhibited a more extended survival trend than the low‐SOCS7 group (20.0 months vs.15.67; hazard ratio, 1.50; 95% CI: 0.98–2.30; *p* = 0.0643) (Figure S6). These findings support our hypothesis that miR‐199a‐3p exerts a negative effect on the survival of patients with PDAC by suppressing the expression of SOCS7 in tumor tissues.

### 3.9 Diagnostic and Survival Analysis of Circulating Exosomal miR‐199a‐3p in Patients With PDAC

We assessed the diagnostic and prognostic potential of miR‐199a‐3p in serum exosomes from 71 individuals, including 10 HDs and 61 patients with PDAC with varying UICC tumor stages. The patients' characteristics are shown in Table S1. Exosomal miR‐199a‐3p expression exhibited good accuracy in distinguishing patients with PDAC from HDs (AUC = 0.827, *p* < 0.001), with a sensitivity of 0.887 and specificity of 0.70 (Figure 4B). Similarly, exosomal miR‐199a‐3p expression showed good accuracy in differentiating stage I/II cases from HDs (AUC = 0.80, *p* = 0.004) (Figure S7).

MiR‐199a‐3p expression in serum exosomes was significantly higher in patients with PDAC than in HDs (*p* < 0.001) (Figure 4C). Moreover, miR‐199a‐3p showed significant differentiation between HD samples and PDAC stages I + II and IV (*p* < 0.05 and *p* < 0.01, respectively; Figure 4D).

We also conducted a survival analysis of 49 patients with PDAC with UICC stage III/IV who underwent chemotherapy. The median observation period for all patients was 12.73 months. The high‐miR‐199a‐3p group exhibited a significantly shorter median PFS than the low‐miR‐199a‐3p group (2.1 vs. 6.5 months; HR = 3.02, 95% CI: 1.52–6.01, *p* = 0.001) (Figure 4E). The high‐miR‐199a‐3p group had a significantly shorter median OS than the low‐miR‐199a‐3p group (4.3 vs. 19.9 months; HR = 3.27, 95% CI: 1.39–7.71, *p* = 0.006) (Figure 4F).

We used a Cox proportional hazards model to analyze the OS of patients with PDAC based on dichotomized factors. Univariate survival analysis indicated that no distant metastasis (*p* = 0.04) and low‐miR‐199a‐3p expression (*p* = 0.01) were potentially favorable prognostic factors. Multivariate survival analysis identified low‐miR‐199a‐3p expression (*p* = 0.01) as an independent prognostic factor for PDAC, favoring a prolonged OS (Table 1).

**TABLE 1:** Univariate and multivariate analysis of the overall survival of patients with PDAC.

Characteristics	Univariate analysis			Multivariate analysis		
	HR	95% CI	*p*	HR	95% CI	*p*
Prognostic cohort (*n* = 49)						
Age (≥ 60 vs. < 60 years)	1.17	0.33–4.13	0.81			
Gender (female vs. male)	1.04	0.42–2.57	0.94			
T1 + 2 vs. T3 + 4 (UICC)	0.86	0.31–2.40	0.77	0.83	0.27–2.57	0.75
Lymph node metastasis (presence vs. none)	2.28	0.85–6.07	0.10	1.87	0.66–5.32	0.24
Distant metastasis (presence vs. none)	4.70	1.06–20.85	0.04	3.63	0.65–20.27	0.14
CA19‐9 (≥ 37 vs. < 37 U/mL)	2.75	0.36–20.82	0.33	2.28	0.24–22.06	0.48
miR‐199a‐3p (high vs. low)	3.49	1.32–9.23	0.01	4.40	1.41–13.77	0.01

Abbreviations: CA 19–9, carbohydrate antigen 19–9; CI, confidence interval; HR, hazard ratio; PDAC, pancreatic ductal adenocarcinoma; UICC, Union for International Cancer Control.

## 4 Discussion

In this study, we demonstrated that exosomal miR‐199a‐3p secreted from CAAs contributes to the malignant transformation of pancreatic cancer cells by promoting the expression of SAA1 via the miR‐199a‐3p/SOCS7/STAT3 pathway. Furthermore, we found that the expression of exosomal miR‐199a‐3p in the serum of patients with PDAC was significantly higher than in the serum of healthy individuals. These findings suggest that miR‐199a‐3p is a promising therapeutic target and a novel biomarker for pancreatic cancer.

Previously, we found that CAA‐CM derived from mouse 3T3 adipocytes promotes the malignant transformation of pancreatic cancer cells via SAA1 upregulation [16]. In the current study, we confirmed that CAA‐CM from human adipocytes also upregulated the expression of SAA1 in pancreatic cancer cells. We observed that CAA‐CM significantly promoted cancer cell proliferation, migration/invasion, and chemoresistance compared to treatment with A‐CM and control media, suggesting that specific CAA‐secreted factors in CAA‐CM promote the malignant transformation of pancreatic cancer cells.

Using exosome labeling and tracking assays, we found that exosomes secreted from CAA were actively taken up by pancreatic cancer cells. Hence, we focused on exosomal miRNAs that regulate SAA1 expression through crosstalk between CAA and pancreatic cancer cells in the tumor microenvironment. Comprehensive RNA‐seq analysis revealed that the expression of miR‐199a‐3p was significantly higher in CAA‐CMs than in A‐CMs. We also confirmed that the transfection of miR‐199a‐3p into Panc‐1 cells increased SAA1 expression and promoted their proliferation, invasion, migration, and gemcitabine resistance. These findings indicated that exosomal miR‐199a‐3p directly contributes to pancreatic cancer progression. Furthermore, considering the significantly elevated expression of miR‐199a‐3p in Panc‐1 cells co‐cultured with CAA‐CM compared to those co‐cultured with A‐CM or a control medium, it is evident that the increase in miR‐199a‐3p expression in Panc‐1 cells was not the result of endogenous synthesis, but rather a direct transfer via CAA‐derived exosomes.

Studies have demonstrated that miR‐199a‐3p can function either as an oncogene or a tumor suppressor in various cancers and could serve as a diagnostic and prognostic biomarker, as well as a potential therapeutic target [29]. For example, miR‐199a‐3p expression is upregulated in gastric cancer tissues, promoting cancer cell proliferation [30] and metastasis [31], and is related to poor prognosis [32]. Similarly, a high‐miR‐199a‐3p expression in colorectal cancer is associated with more advanced disease [33] and shorter OS [34]. MiR‐199a‐3p expression was also reported to be induced in patient‐derived pancreatic CAFs and serves as a key regulator of their tumor‐promoting effects [35]. Since CAAs are thought to be one of the origins of CAFs [13, 14, 16] and that ISH of pancreatic cancer tissues showed increased miR‐199a‐3p expression in CAAs and tumor cells (Figure S4), these results corroborate our hypothesis that CAAs play a vital role in the secretion of miR‐199a‐3p in the pancreatic cancer microenvironment.

Notably, miR‐199a‐3p has been implicated in adipocyte differentiation through the regulation of the mTOR pathway [36], stearoyl‐CoA desaturase signaling [37], and KDM6A/WNT signaling [38]. Furthermore, miR‐199a‐3p expression is modulated by fatty acids and inflammatory factors during adipogenesis [39]. These findings indicate that miR‐199a‐3p plays a pivotal role in the dedifferentiation of adipocytes into CAAs; however, further clarification is needed.

We hypothesized that miR‐199a‐3p induces SAA1 expression by activating STAT3, as STAT3 reportedly interacts with the *SAA* promoter [40, 41, 42]. STAT3 is a transcription factor that regulates the expression of various genes involved in critical cellular processes, including cell growth, apoptosis, inflammation, and the immune response [43]. Recent studies have revealed that STAT3 acts downstream of the NF‐κB‐responsive elements in the *SAA1* promoter region via a cis‐acting mechanism [40, 41] and plays an essential role in cytokine‐driven SAA expression [42]. However, TargetScan database analysis revealed that miR‐199a‐3p does not directly bind to STAT3. Hence, we searched for target genes that mediate the interaction between miR‐199a‐3p and STAT3. With reference to the report that miR199a‐3p repressed SOCS7 expression resulting in STAT3 activation during human renal fibrosis [44], the luciferase reporter assays in the present study confirmed that miR‐199a‐3p directly binds to *SOCS7*. SOCS7 is a member of the suppressors of cytokine signaling family that represses STAT3 activity by interacting with phosphorylated STAT3 [45]. We confirmed that miR‐199a‐3p‐mediated SOCS7 silencing significantly enhanced STAT3 phosphorylation in three pancreatic cancer cell lines. This was supported by previous studies that found SOCS7 silencing promoted the nuclear translocation of STAT3 in bladder cancer cells [46].

**FIGURE 5:**
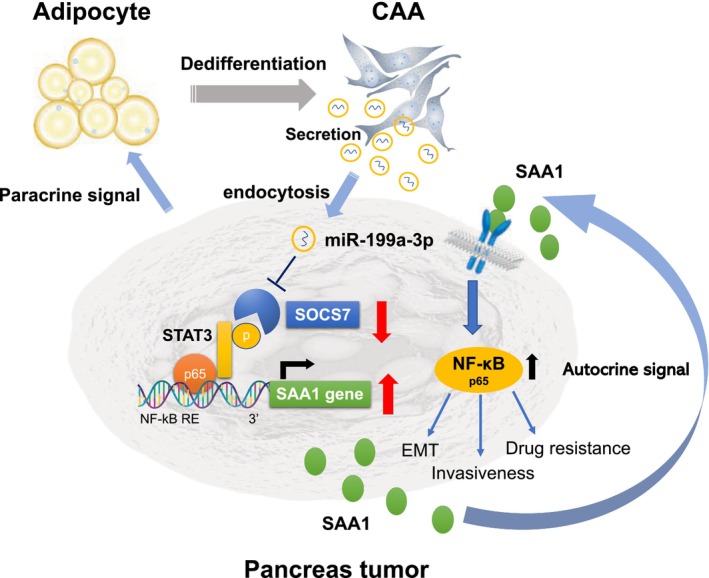
Proposed model illustrating the role of CAA‐derived exosomal miR‐199a‐3p in regulating pancreatic cancer. Adipocytes in contact with pancreatic cancer cells are first dedifferentiated into cancer‐associated adipocytes (CAAs) in the microenvironment of pancreatic cancer. Subsequently, exosomal miR‐199a‐3p secreted from CAAs are taken up by surrounding pancreatic cancer cells, then directly bind to the 3′‐untranslated region (UTR) of SOCS7 and suppress its expression, which in turn activates STAT3 and increases SAA1 expression. The secreted SAA1 then promotes pancreatic cancer cell proliferation, metastasis, and chemoresistance by activating NF‐κB, suggesting that miR‐199a‐3p plays a vital role as a mediator of the microenvironment in pancreatic cancer.

We postulated that adipocytes in contact with pancreatic cancer cells undergo dedifferentiation into CAAs within the tumor microenvironment. Subsequently, exosomal miR‐199a‐3p secreted from CAAs and taken up by surrounding pancreatic cancer cells suppressed SOCS7 expression, leading to STAT3 activation and increased SAA1 expression, ultimately resulting in a more aggressive phenotype in pancreatic cancer cells. Moreover, secreted SAA1 can act on neighboring pancreatic cancer cells, promoting their metastatic potential and chemoresistance via NF‐κB activation [16]. These findings suggested that miR‐199a‐3p plays a crucial role as a mediator in establishing an autocrine/paracrine loop in the pancreatic cancer microenvironment (Figure 5).

Liquid biopsy has gained popularity for cancer detection using blood samples, and exosomes have been identified as specific biomarkers for liquid biopsies, including pancreatic cancer [47]. Therefore, we examined the clinical significance of miR‐199a‐3p in diagnosing and predicting the malignant potential of PDAC. Our study revealed that miR‐199a‐3p expression was elevated in the serum of patients with PDAC compared to that of healthy individuals, indicating its potential association with CAA induction during PDAC progression. Exosomal miR‐199a‐3p showed a relatively high diagnostic value (AUC = 0.827) for PDAC, differentiating it from non‐PDAC patients.

Moreover, high miR‐199a‐3p expression was significantly associated with poor OS and PFS in our clinical data and TCGA database, indicating its correlation with a poor prognosis. Furthermore, multivariate analysis confirmed that miR‐199a‐3p exosomes are potential independent prognostic markers of PDAC. Although this study was conducted retrospectively with a limited number of patients, its findings suggest that serum exosomal miR‐199a‐3p has prognostic and diagnostic potential in PDAC. Therefore, further research is warranted to investigate the utility of miR‐199a‐3p as a biomarker of PDAC in a larger prospective cohort.

Recent studies suggest that miRNAs delivered through exosomes can be used as anticancer therapeutic tools because of their intrinsic properties [23, 24]. We demonstrated that crosstalk between adipocytes and cancer cells induces CAAs upon close proximity between the two cell types; however, since exosomes can travel through the circulation, CAA‐derived exosomal miRNAs can communicate with proximal and distant metastatic lesions. Thus, new therapeutic strategies, such as blocking the function of exosomal miR‐199 secreted by CAAs, could be used to treat primary lesions, metastases, and chemoresistant tumors. Although the mechanism by which pancreatic cancer enhances the expression of miR‐199a‐3p in adipocytes during adipogenic dedifferentiation remains to be elucidated, a better understanding of the mechanism underlying CAA induction in pancreatic cancer may help develop promising therapeutic targets and approaches for CAA‐related cancer therapy.

In conclusion, our study highlights the role of CAAs in promoting pancreatic cancer progression through the secretion of miR‐199a‐3p‐enriched exosomes targeting the SOCS7/STAT3/SAA1 pathway. Additionally, we suggest that serum exosomal miR‐199a‐3p may serve as a potential diagnostic and prognostic biomarker for PDAC.

## Author Contributions

**Kazuyoshi Noda:** conceptualization (equal), data curation (equal), investigation (equal), methodology (equal), writing – original draft (equal). **Yasushi Sato:** conceptualization (lead), data curation (equal), funding acquisition (lead), investigation (lead), methodology (lead), writing – original draft (lead), writing – review and editing (lead). **Yasuyuki Okada:** data curation (equal), investigation (equal), methodology (equal). **Kensei Nishida:** data curation (equal), investigation (equal). **Yutaka Kawano:** investigation (equal), methodology (equal). **Toshihito Tanahashi:** formal analysis (equal), methodology (equal). **Masahiro Bando:** formal analysis (supporting), methodology (supporting). **Koichi Okamoto:** investigation (supporting). **Masanori Takehara:** investigation (supporting). **Masahiro Sogabe:** investigation (supporting). **Hiroshi Miyamoto:** investigation (supporting). **Kei Daizumoto:** investigation (supporting), methodology (equal). **Hiroomi Kanayama:** investigation (supporting), methodology (supporting). **Tetsuji Takayama:** conceptualization (lead), funding acquisition (equal), investigation (equal), methodology (equal), writing – review and editing (equal).

## Ethics Statement

This study was conducted in accordance with the principles of the Declaration of Helsinki. Written informed consent was obtained from all patients, and the study was approved by the Institutional Review Board of Tokushima University (approval numbers: 3420, 2222).

## Conflicts of Interest

The authors declare no conflicts of interest.

## Supporting information


Data S1.

## Data Availability

All data generated or analyzed during this study are included either in this article or in additional files, and the links to public datasets are provided in the manuscript.
