# Supplementary material for: Exosomal miR‐199a‐3p Secreted From Cancer‐Associated Adipocytes Promotes Pancreatic Cancer Progression
Source: Cancer Med. 2024 Oct 21;13(20):e70265. doi: 10.1002/cam4.70265 (PMC11492146; doi:10.1002/cam4.70265)
Supplement: Supplementary file 1 — Data S1. [file CAM4-13-e70265-s001.docx]

# Supplemental information

**Exosomal miR-199a-3p secreted from cancer-associated adipocytes promote pancreatic cancer progression**

Kazuyoshi Noda^1^, Yasushi Sato^2^, Yasuyuki Okada^1^, Kensei Nishida^3^, Yutaka Kawano^1^, Toshihito Tanahashi^1^, Masahiro Bando^1^, Koichi Okamoto^1^, Masanori Takehara^1^, Masahiro Sogabe^1^, Hiroshi Miyamoto^1^, ﻿Kei Daizumoto^4^, Hiroomi Kanayama^4^, Tetsuji Takayama^1^

^1^Department of Gastroenterology and Oncology, Tokushima University Graduate School of Biomedical Sciences, Tokushima, Japan.

^2^Department of Community Medicine for Gastroenterology and Oncology, Tokushima University Graduate School of Biomedical Sciences, Tokushima, Japan

﻿^3^Department of Pathophysiology, Tokushima University Graduate School of Biomedical Sciences, Tokushima, Japan.

^4^Department of Urology, Tokushima University Graduate School of Biomedical Sciences, Tokushima, Japan.

**Corresponding author:** Tetsuji Takayama, M.D., Ph.D., 3-18-15, Kuramoto-cho,

Tokushima, 770-8503, Japan. Tel: +81-88-633-7124. Fax: +81-88-633-9235.

E-mail: [takayama@tokushima-u.ac.jp](mailto:takayama@tokushima-u.ac.jp)

**Supplementary** **information**

**Methods**

**Transmission electron microscopy**

**﻿**For transmission electron microscopy (TEM), exosomes were suspended in glutaraldehyde, dropped onto carbon-coated 400 mesh copper grids, and stained with 2% uranyl acetate. After drying, the samples were imaged using an H-7600 TEM (Hitachi) at 100 kV.

**Western blot analysis**

Protein expression was analyzed using western blotting as previously described [26]. Cells or exosomes were lysed in RIPA lysis buffer containing a complete protease and phosphatase inhibitor cocktail (Sigma-Aldrich, St Louis, MO, USA). Briefly, 30 mg of protein per sample was loaded onto SDS-PAGE gels and then transferred onto polyvinylidene fluoride membranes using a semidry transfer apparatus (Bio-Rad, Hercules, CA, USA). The membranes were then blocked and incubated with primary antibodies at 4°C overnight, followed by incubation with the corresponding horseradish peroxidase-conjugated secondary antibodies at room temperature. Finally, the membranes were visualized with a Thermo Pierce chemiluminescent (ECL) Western Blotting Substrate (Thermo Fisher Scientific, Waltham, MA, USA) using a Tanon 5200 system (Tanon, Shanghai, China). The following primary antibodies were used for western blotting: anti-SOCS7 (1:1,000; Abcam, Cambridge, UK), anti-STAT3 (1:1,000; Cell Signaling Technology, Danvers, MA, USA; RRID: AB_331757), anti-phospho-STAT3 (1:2,000; Cell Signaling Technology, Danvers, MA, USA; RRID: AB_2491009), anti-CD63 (1:200; Santa Cruz, Dallas, TX, USA; RRID: AB_627877), anti-Alix (1:200; Santa Cruz, Dallas, TX, USA; RRID: AB_673819), anti-tsg101 (1:200; Santa Cruz, Dallas, TX, USA; RRID: AB_671392), and anti-β-actin (1:20,000; Sigma-Aldrich, St Louis, MO,USA; RRID: AB_476744).

**Exosome labeling and tracking**

Purified exosomes were labeled using a PKH26 Red Fluorescent Cell Linker Kit (Sigma-Aldrich: PKH26GL, MO, USA) according to the manufacturer’s instructions. Briefly, the exosomes were centrifuged at 14,000 ×*g* for 2 min using an Amicon Ultra 0.5 mL 100K tube (Merck). Subsequently, the labeled exosome pellets were resuspended and added to Panc-1 cells for exosome uptake analysis. After incubation for 24 h, ﻿the slides were fixed in a 4% paraformaldehyde solution. DAPI (Invitrogen, Waltham, MA, USA) was used for nuclear staining. Finally, the slides were observed under a fluorescence microscope (Keyence: BZ-X710, Osaka, Japan).

**Small interfering RNA transfection**

Cells were transfected with 10 nM siRNAs against STAT3 (Silencer Select siRNA STAT3, 4390824; Assay ID s745, Thermo Fisher Scientific) or scramble siRNAs (Silencer Select Negative Control No.1 siRNA, 4390843) using Lipofectamine™ RNAiMAX Transfection Reagent (Thermo Fisher Scientific) in Opti-MEM medium. STAT3 gene knockdown was confirmed by real-time polymerase chain reaction (PCR) using the TaqMan Gene Expression assay for STAT3.

## **Proliferation assays**

**﻿**Cell proliferation was analyzed using a cell counting kit (CCK-8; Dojindo) according to the manufacturer’s instructions. Briefly, the cells were seeded in 96-well plates (3×10^3^ cells/well) and incubated for 24 h at 37°C. Dulbecco’s modified Eagle’s medium (DMEM), adipocyte-conditioned medium (A-CM), and cancer-associated adipocyte-conditioned medium (CAA-CM) were added to the wells, and the plates were incubated for another 72 h at 37°C. CCK-8 solution was then added to the wells, and the plates were incubated for 3 h at 37°C. The absorbance of the wells at 450 nm was determined using a Spectra Max i3x platform (Molecular Devices, Inc., Danaher Corporation). The IC_50_ values were determined using nonlinear regression analysis.

## ﻿**Cell migration assay**

Cell migration assays were performed using a CytoSelect 24-Well Cell Invasion Assay Kit (CELL BIOLABS: CBA-110). Panc-1 cells were plated in the upper chamber, and DMEM, A-CM, and CAA-CM were added to the lower chamber. After 48 h of culture, the cells were stained with 0.1% crystal violet for 30 min, and non-migrating or non-invading cells were removed. Six visual fields were selected randomly to calculate the number of migrating cells.

## **Scratch wound healing assay**

Panc-1 cells (1.0×10^6^ cells/well) were seeded into 6-well plates and incubated with DMEM. After the cultures reached 90% confluency, the center of the wells was scratched with a 200-μL pipette tip, and the spent media were changed to DMEM, A-CM, or CAA-CM. After 24 h, wound healing was imaged using a BZ-X710 fluorescence microscope (Keyence). The wound healing ability was determined by measuring the mean migration distance, which was calculated by dividing the wound repair area by the width.

## **Drug sensitivity assay**

The drug sensitivity of the cells was determined using the CCK-8 assay (Dojindo), according to the manufacturer’s instructions. In brief, the cells plated in 96-well plates were cultured with A-CM, CAA-CM, or DMEM for 24 h and then treated with different concentrations of gemcitabine ranging from 0.1 to 100 μM for another 72 h. The absorbance of each well was measured at 450 nm after incubation with a CCK-8 solution (Dojindo). The IC_50_ values were determined via nonlinear regression analysis using GraphPad Prism software (v8.0, San Diego, CA, USA).

**miRNA sequencing**

Exosomes were isolated from three pairs of A-CM and CAA-CM from three donors, following the procedure described earlier. Total RNA was extracted from the purified exosomes using an RNAqueous-Micro Kit (Thermo Fisher Scientific), and the amount and quality of small RNA in the total RNA samples were determined using an Agilent small RNA kit on an Agilent 2100 Bioanalyzer.

Briefly, 5 ng of the small RNAs were subjected to library construction using a SMARTer smRNA-Seq Kit for Illumina (Takara Bio, Shiga, Japan), according to the manufacturer’s protocol. Sequencing libraries were constructed via polyadenylation, cDNA synthesis, and PCR amplification. The resulting cDNA library included the sequences required for clustering in an Illumina flow cell. Subsequently, the libraries were gel-purified and validated by checking their size, purity, and concentration using an Agilent Bioanalyzer. The libraries were then quantified using qPCR according to the qPCR Quantification Protocol Guide (KAPA Library Quantification kits for Illumina Sequencing platforms) and qualified using a TapeStation D1000 ScreenTape (Agilent Technologies, Santa Clara, CA, USA). Subsequently, the libraries were pooled in equimolar amounts and sequenced using an Illumina HiSeq 2500 (Illumina, San Diego, CA, USA) instrument to generate 51-base-reads. Image decomposition and quality value calculations were performed using the Illumina pipeline modules. Sequence alignment and the detection of known and novel miRNAs were performed using miRDeep2 (v2.0.0.8) [1]. Reads with the ribosomal RNA removed were aligned to the reference genome (miRBase v22.1) [2] and non-coding RNA database (RNAcentral 14.0) [3] to classify known miRNAs and other types of RNA. Differentially expressed miRNAs between the two groups were determined using the criteria fold change ≥2 and p-value <0.05. Missing data were abandoned or imputed using the nearest-neighbor method, and the z-score and 0–1 normalization methods were further conducted on the miRNA expression data. Library construction, sequencing reactions, and bioinformatics analysis were performed at Macrogen, Inc. Sequence data were deposited in the Gene Expression Omnibus database with the accession number GSE 230561.

## ﻿**Luciferase reporter gene activity assay**

Double-stranded oligonucleotides for the 3′-UTR of SOCS7 harboring miR-199a-3p

binding sites were prepared by heating equal amounts of complementary single strands at 95°C for 15 min and gradually cooling to room temperature. The DNA fragments were then subcloned into the psiCHECK-2 vector (Promega) using the *Xho*I and *Not*I restriction sites. Plasmids with mutated miR-199a-3p binding sites were also prepared by replacing three base pairs at the 3′-UTR of the seed sequence. Panc-1 cells (8.0×10^4^ cells/well) were seeded into 24-well plates. After 24 h of incubation, the cells were transfected with miR-199a-3p (10 nM) or the control mimic miRNA (10 nM) using Lipofectamine 3000 reagent (Thermo Fisher Scientific). Twelve hours after transfection with the mimic miRNAs, psiCHECK-2 constructs with various site-directed mutations were transfected into the cells. Eight hours after the second transfection, the cells were harvested, and the firefly and Renilla luciferase activities were measured using a Dual-Luciferase Reporter Assay System (Promega). Luciferase activity in each cell lysate was measured using a Fluostar Galaxy plate reader (BMG Lab Technologies). Relative luciferase activity was normalized to Renilla luciferase activity and compared with those of the respective controls.

## ***In situ* hybridization (ISH) of miR-199a-3p**

miRNA ISH was performed on formalin-fixed and paraffin-embedded tissue sections using a miRNAscope HD Assay (Advanced Cell Diagnostics) according to the manufacturer’s instructions. Briefly, sections were deparaffinized in xylene and rehydrated with graded ethanol, followed by a final wash in phosphate buffered saline (PBS). The sections were then incubated with proteinase K and hybridized with miRNAscope™ Probe - SR-hsa-miR-199a-3p-S1 probe. A specific anti-DIG antibody directly conjugated to alkaline phosphatase was then applied, and the sections were incubated in KTBT buffer. The slides were counterstained with hematoxylin. The miRNAscope™ Negative Control Probe-SR-Scramble-S1 was used as a control.

**Immunohistochemistry**

Immunohistochemical staining for SOCS7 and pSTAT in paraffin-embedded sections of pancreatic cancer tissues was performed using the polymer method. In brief, 4-mm sections were dewaxed and rehydrated. Blocking POD is performed by 0.3% H_2_O_2_ -MeOH for 45 min. They were then incubated with an Anti- SOCS7 Polyclonal Antibody (1:100, bs-20151R, Bioss, Boston, MA, USA) or Phospho-STAT3 rabbit monoclonal antibody (1:100, 9145S, Cell Signaling Technology, Danvers, MA, USA) at 4 °C overnight, washed with PBS, and incubated with the secondary antibody ImmPRESS Universal Polymer Kit (MP-7500, Vector, Newark, CA, USA) at room temperature for 60 min. Next, incubation was performed in a chromogenic solution prepared with DAB (3-3’-diaminobenzidine) for 2.5 min in a dark chamber, and hematoxylin was used for counterstaining. Rabbit mAb IgG Isotype Control (1:100, 3900S, Cell Signaling Technology, Danvers, MA, USA) was used as a positive control.

## **﻿Blood sample collection**

Blood samples were collected before the initiation of treatment. After collection, the blood samples were centrifuged at 2000 × *g* for 15 min at 4°C. The clear upper layer comprising the cell-free serum was extracted and stored at −80°C until use.

## **RNA extraction and quantitative RT-qPCR analysis**

## Exosomes were separated from serum by ultracentrifugation, as described in the 'Methods' section, and total RNA was extracted using the miRNeasy Micro kit (Qiagen) according to the manufacturer's instructions. For miRNA, complementary DNA was synthesized from total RNA using a TaqMan microRNA Reverse Transcription Kit (Thermo Fisher Scientific). RT-qPCR analysis was performed using MicroRNA Assay Kits and TaqMan Universal Master Mix II, no UNG (Applied Biosystems) on a QuantStudio3 3 Flex Real-Time PCR System (Applied Biosystems). Gene expression levels were evaluated using QuantStudio 3 Flex Real-Time PCR System Software (Applied Biosystems). The relative expression level of miR-199-3p was evaluated and normalized to that of U6 (internal control) using the 2^-ΔΔCt^ method (Fig.S8) Normalized expression values were log_2_-transformed. The assay IDs of hsa-miR-199-3p and U6 used in this study were 002304 and 001973, respectively.

**Reference**:

1. Friedländer MR, MacKowiak SD, Li N, et al. MiRDeep2 accurately identifies known and hundreds of novel microRNA genes in seven animal clades. *Nucleic Acids Res* 2012;40:37–52. <https://doi.org/10.1093/nar/gkr688>.
2. Kozomara A, Griffiths-Jones S. MiRBase: Annotating high confidence microRNAs using deep sequencing data. *Nucleic Acids Res* 2014;42:D68–73. <https://doi.org/10.1093/nar/gkt1181>.
3. Petrov AI, Kay SJE, Kalvari I, *et al.* RNAcentral: A comprehensive database of non-coding RNA sequences. *Nucleic Acids Res* 2017;45:D128–34. <https://doi.org/10.1093/nar/gkw1008>.

# Figure S1

**Figure S1. Cell proliferation, migration/invasion, and drug resistance in Panc-1 cells cultured with the conditioned medium (CM) from cancer-associated adipocytes** **(CAAs). A,** Phenotypic change from adipocytes to CAAs following co-culture with Panc-1 cells. Representative images of mature adipocytes established from patients with benign disease (upper panel) and adipocytes co-cultured with Panc-1 cells (lower panel) stained with Oil Red O. Scale bar, 100 µm. **B,** Lipid levels were determined by extracting Oil Red O with isopropanol and measuring the absorbance at 496 nm (n=3). **C,** ﻿Proliferation of Panc-1 cells cultured with CAA-CM, adipocyte (A)-CM, or the control medium was analyzed using the WST-8 assay; n=6. *p<0.05. **D–E,** The migration area in Panc-1 cells cultured with A-CM, CAA-CM, or the control medium was assessed using a wound healing assay (**D**). The migration area was quantified using TScratch software (**E**). n=6. Scale bars, 200 µm. **F–G,** Invasion capability of Panc-1 cells cultured with A-CM, CAA-CM, or the control medium was assessed using a Transwell invasion assay. Representative images of invading cells stained using a cell staining solution (**F**)**.** The migrated cells were quantified by counting them in six randomly selected visual fields (**G**). n=6. Scale bar, 200 µm. **H,** Panc-1 cells seeded in 96-well plates were cultured in A-CM, CAA-CM, or the control medium for 72 h. The cells were then treated with different concentrations of gemcitabine for 72 h, and the number of viable cells was quantified using a WST assay. The IC_50_ values were determined using nonlinear regression analysis (n=6). **I,** Relative *SAA1* mRNA expression in Panc-1 cells co-cultured with A-CM, CAA-CM, or the control medium for 72 h. n=6. *p<0.05

**Figure S2**

**Figure S2.** Relative miR-199a-3p levels in Panc-1 cells transfected with miR-199a-3p mimics or control miRNA as determined using RT-qPCR. n=6. *p<0.05.

**Figure S3**

**Figure S3. Cell proliferation, migration/invasion, and drug resistance in Panc-1 cells transfected with siSTAT3. A,** ﻿Proliferation of Panc-1 cells transfected with miRNA199+siNC, miRNA199+siSTAT3, siSTAT3, or the controls was analyzed using the WST-8 assay; n=6. *p<0.05. **B,** The migration area in Panc-1 cells transfected with miRNA199+siNC, miRNA199+siSTAT3, siSTAT3, or the controls was assessed using a wound healing assay. The migration area was quantified using TScratch software. n=6. Scale bars, 200 µm. **C,** Invasion capability of Panc-1 cells transfected with miRNA199+siNC, miRNA199+siSTAT3, siSTAT3, or the controls was assessed using a Transwell invasion assay. Representative images of invading cells stained using a cell staining solution. The migrated cells were quantified by counting them in six randomly selected visual fields. n=6. Scale bar, 100 µm. **D,** Panc-1 cells seeded in 96-well plates were transfected with miRNA199+siNC, miRNA199+siSTAT3, siSTAT3, or the controls and cultured for 72 h. The cells were then treated with different concentrations of gemcitabine for 72 h, and the number of viable cells was quantified using a WST assay. The IC_50_ values were determined using nonlinear regression analysis (n=6). **E**, Relative STAT levels in Panc-1 cells transfected with siSTAT or control siRNA as determined using reverse transcription-quantitative polymerase chain reaction (RT-qPCR). n=6. *p<0.05. **F**, Relative miR-199a-3p levels in Panc-1 cells transfected with miR-199a-3p mimics or control miRNA as determined using RT-qPCR. n=6. *p<0.05.

**Figure S4**

**Figure S4. *In situ* hybridization of miR-199a-3p in human pancreatic cancer tissues.** Representative images from consecutive sections of human pancreatic cancer tissues stained using an miR-199a-3p probe (a, b, c) or a negative control scramble probe (d, e, f) are shown. Magnified images (5×, 20×, and 40× magnification) of the boxes in panels a and d are displayed. Strong miR-199a-3p staining in CAAs and tumor cells was observed around the tumor-stroma interface. Digoxigenin-labeled miR-199a-3p probes were detected using anti-digoxigenin-alkaline phosphatase. Sections were counterstained with hematoxylin. CAAs, cancer-associated adipocytes.

**Figure S5**


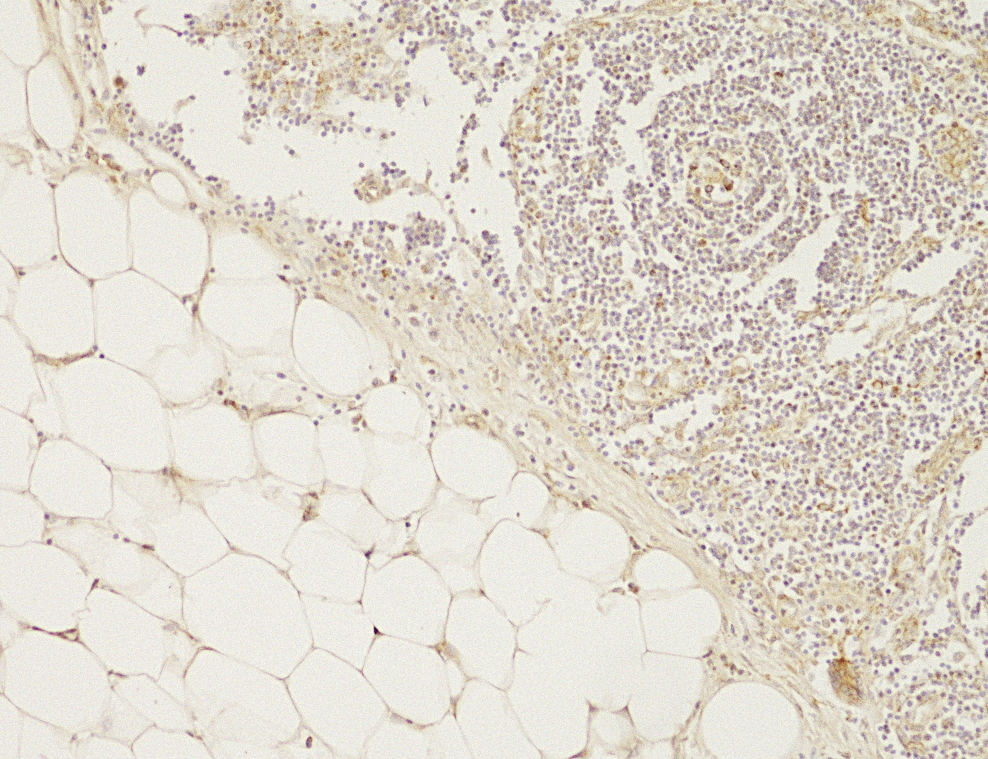


**
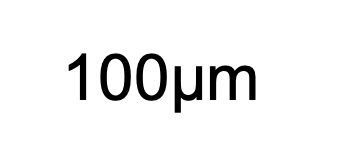
**

**﻿** **Figure S5. In situ hybridization of miR-199a-3p and immunohistochemistry of SOCS7 and pSTAT in human pancreatic cancer tissues.** Representative images of consecutive sections of human pancreatic cancer tissues stained using an miR-199a-3p probe and anti-SOCS7 and anti-pSTAT antibodies are shown. Magnified images (40× magnification) of the left panels are shown in the right panels. Arrows indicate the nuclear expression of pSTAT3. The sections were counterstained with hematoxylin.

**Figure S6**

**Figure S6. Kaplan–Meier survival curves showing the overall survival of the low-SOCS7 and high-SOCS7 groups from the GDC TCGA Pancreatic Cancer (PAAD) dataset.** The log_2_(x + 1) transformed RNA-Seq by Expectation-Maximization (RSEM) normalized counts were used to perform survival analyses. The cutoff value was set at 5.1413 based on the median RNA expression. Cases with ≥5.1413 and <5.1413 were designated the high and low expression groups, respectively.

**Figure S7**

**Figure S7. Exosomal miR-199a-3p expression showed good accuracy in differentiating stage I/II cases from HD.** Receiver operating characteristic (ROC) curve analysis for discriminating between healthy individuals (n=10) and patients with stages I + II (n=12) pancreatic ductal adenocarcinoma (PDAC).

**Figure S8.**

**Figure S8. The relative expression level of U6 (internal control) using the 2^-ΔΔCt^ method.** The expression of U6 in the samples obtained in this study showed little variation among the samples (p=0.655, healthy donors vs. PDAC patients).

**Table S1**Patients’ characteristics.

| **Category** | **Total** | **Healthy**  **controls** | **PDAC**  **UICC I** | **PDAC**  **UICC II** | **PDAC**  **UICC III** | **PDAC**  **UICC IV** |
| --- | --- | --- | --- | --- | --- | --- |
| Number of  patients | 71 | 10 | 11 | 1 | 13 | 36 |
| **Age (years)** | | | | | | |
| Median  (range) | 69 (33-87) | 60 (45-64) | 73 (56-87) | 68 | 70 (62-81) | 67.5 (33-87) |
| <60 | 10 | 2 | 1 | 0 | 0 | 7 |
| ≥60 | 61 | 8 | 11 | 1 | 13 | 29 |
| **Gender** | | | | | | |
| Female | 38 | 5 | 8 | 0 | 7 | 18 |
| Male | 33 | 5 | 3 | 1 | 6 | 18 |
| **Serum CA.19-9 (U/mL)** | | | | | | |
| Median  (range) |  |  | 44  (1-8265) | 315 | 364  (1-33090) | 5679.55  (19-1925228) |

PDAC, pancreatic ductal adenocarcinoma; UICC, Union for International Cancer Control; CA.19-9, carbohydrate antigen 19-9

**Table S2** Characterization of A-CM/CAA-CM-derived exosomes.

|  | particle diameter (nm) | concentration (/mL) |
| --- | --- | --- |
|  | Mean |  |
| A-CM | 81 - 171 | 2.14E+11 - 1.96E+12 |
| CAA-CM | 109 - 128 | 4.28E+11 - 8.36E+11 |

Adipocyte-derived conditioned medium (A-CM), Cancer-associated adipocyte-derived conditioned medium (CAA-CM)
